# Lake water chemistry and local adaptation shape NaCl toxicity in *Daphnia ambigua*

**DOI:** 10.1111/eva.13668

**Published:** 2024-03-22

**Authors:** Mary A. Rogalski, Elizabeth S. Baker, Clara M. Benadon, Christoph Tatgenhorst, Brady R. Nichols

**Affiliations:** ^1^ Bowdoin College Brunswick Maine USA

**Keywords:** *Daphnia*, ecotoxicology, freshwater salinization, local adaptation, road salt, water hardness

## Abstract

The increasing application of road deicing agents (e.g., NaCl) has caused widespread salinization of freshwater environments. Chronic exposure to toxic NaCl levels can impact freshwater biota at genome to ecosystem scales, yet the degree of harm caused by road salt pollution is likely to vary among habitats and populations. The background ion chemistry of freshwater environments may strongly impact NaCl toxicity, with greater harm occurring in ion‐poor, soft water conditions. In addition, populations exposed to salinization may evolve increased NaCl tolerance. Notably, if organisms are adapted to the water chemistry of their natal environment, toxicity responses may also vary among populations in a given test medium. We examined the potential for this evolutionary and environmental context to interact in shaping NaCl toxicity with a pair of laboratory reciprocal transplant toxicity experiments, using natural populations of the water flea *Daphnia ambigua* collected from three lakes that vary in ion availability and composition. We observed a strong effect of the lake water environment on NaCl toxicity in both trials. NaCl caused a much greater decline in reproduction and *r* in lake water from a low‐ion/calcium‐poor environment (20 μS/cm specific conductance; 1.7 mg/L Ca^2+^) compared with water from both a Ca^2+^‐rich lake (55 μS/cm; 7.2 mg/L Ca^2+^) and an ion‐rich coastal lake (420 μS/cm; 3.4 mg/L Ca^2+^). *Daphnia* from this coastal lake were most robust to the effects of NaCl on reproduction and *r*. A significant interaction between the population and lake water environment shaped survival in both trials, suggesting that local adaptation to the test waters used may have contributed to toxicity responses. Our findings that the lake water environment, adaptation to that environment, and adaptation to a contaminant of interest may shape toxicity demonstrate the importance of considering environmental and biological complexity in mitigating pollution impacts.

## 1 INTRODUCTION

The salinization of freshwater environments is an emerging water quality concern in regions around the world (Dugan et al., 2017; Kaushal et al., 2018, 2021). A key contributor to this increase in salinity is the application of rock salt (NaCl) as a road deicing agent (Novotny & Stefan, 2010). In the snow‐belt regions of the United States upwards of 22–25 million metric tons of NaCl are applied to highways annually (Bolen, 2022), with the consequence of climbing concentrations of chloride in streams, rivers, lakes, and ponds (Kaushal et al., 2005, 2018). While research examining ecological impacts of freshwater salinization lags behind observations of widespread changes in water chemistry, studies show that increased salinity can impact freshwater systems at genome to ecosystem scales (Griffith, 2017; Hairston et al., 2005; Kipriyanova et al., 2007; Latta et al., 2012; Lind et al., 2018; Michels et al., 2003; Searle et al., 2016; Van Meter et al., 2011; Weider & Hebert, 1987).

Chronic exposure to elevated NaCl causes a number of sublethal effects including suppressed feeding rates, decreased respiration and reduced growth, reduced fecundity and reproductive delay, as well as behavioral and developmental abnormalities (Sanzo & Hecnar, 2006; Stoks et al., 2014; Venâncio et al., 2018). Such impacts on development and life history may drive population level impacts of NaCl exposure (Sarma et al., 2006; Searle et al., 2016). Interspecific variation in salinity tolerance (Elphick et al., 2011; Heine‐Fuster et al., 2010; Sarma et al., 2006) and changing species interactions (Coldsnow, Relyea, et al., 2017; Hall et al., 2013; Hintz & Relyea, 2017) may explain observations of decreased biodiversity, changes in community composition, and altered trophic dynamics in salinized freshwater environments (Arnott et al., 2020; Gutierrez et al., 2018; Hairston et al., 2005; Jensen et al., 2010; Kipriyanova et al., 2007; Sinclair & Arnott, 2018; Stoler et al., 2017; Van Meter et al., 2011; Wu et al., 2006). Halophilic species may even colonize recently salinized environments far from their typical species range (Hairston et al., 2005; Kipriyanova et al., 2007). In addition, intraspecific genetic variation in sea salt exposure has been linked to microgeographic adaptation (Richardson et al., 2014) to NaCl stress and even divergence in osmoregulatory strategies (Weider & Hebert, 1987).

The U.S. Environmental Protection Agency established a chronic water quality criterion of 230 mg/L chloride based on laboratory tests of NaCl exposure on vertebrate and invertebrate test species (Benoit & Stephan, 1988). Examination of multidecadal trends in water quality of hundreds of lakes in the US suggests that many temperate lakes could exceed this EPA threshold for chronic chloride exposure within 50 years (Dugan et al., 2017); yet, modeling work exploring landscape patterns of road salt use and inputs from watersheds indicates equilibrium chloride concentrations below 230 mg/L could be achievable for many US lakes as long as road density and road salt application rates do not increase (Solomon et al., 2023). At the same time, laboratory assays used to establish this 230 mg/L chloride toxicity threshold may not accurately predict chloride impacts in natural environments as they do not account for ways in which environmental and biological complexity might influence toxicity. Evidence that factors such as lake water chemistry (Arnott et al., 2020; Mount et al., 2016) and evolutionary history (Brady, 2012, 2013; Brady, Monosson, et al., 2017; Brady, Richardson, et al., 2017; Coldsnow, Mattes, et al., 2017; Weider & Hebert, 1987) may shape NaCl toxicity warrants further examination to understand how organisms embedded in their natural environment may vary in their susceptibility to road salt pollution.

The background ion chemistry of a freshwater environment may strongly affect the degree to which NaCl pollution causes physiological or ecological impacts. In particular, robust laboratory study supports that NaCl toxicity decreases with increasing water hardness (Elphick et al., 2011; Gillis, 2011; Mount et al., 2016; Soucek et al., 2011, 2018). For example, experimentally increasing water hardness (as CaCO_3_) from 10 to 160 mg/L resulted in an approximately six‐fold decrease in acute chloride toxicity in the model zooplankter *Ceriodaphnia dubia* (Elphick et al., 2011). While both calcium and magnesium contribute to overall water hardness, calcium specifically has been shown to play a key role in mitigating the toxicity of NaCl and other sodium‐based salts (Davies & Hall, 2007; Mount et al., 2016). Calcium may help reduce sodium toxicity due to mutual competition between calcium and sodium ions in ion transport (Ahearn et al., 2001; Griffith, 2017). Low‐calcium concentrations may also increase the permeability of gill epithelia, exacerbating osmoregulatory stress (Brauner et al., 2012; Gundersen & Curtis, 1995). Soft‐water conditions predominate in regions where erosion‐resistant bedrock and poor, sandy soils dominate the landscape (Norton et al., 1989), especially where historic impacts of lake acidification caused by industrial activity exacerbate calcium limitation (Hessen et al., 2017; Jeziorski et al., 2008). Notably, Arnott et al. (2020) observed chronic toxicity of NaCl for *Daphnia* species at concentrations ranging from 5 to 40 mg/L chloride, an order of magnitude lower than the EPA's 230 mg/L recommendation, when tested in artificial lake water designed to mimic soft‐water conditions commonly observed in Canadian Shield lakes (2.54 mg/L Ca^2+^) (Celis‐Salgado et al., 2008). For context, the toxicity trials that contributed to the U.S. EPA's 230 mg/L chloride limit used test waters with 40 mg/L Ca^2+^ (Birge et al., 1985). Based on this body of evidence, toxicological threats of increasing salinization should be expected to vary considerably among freshwater bodies depending on the background ionic concentrations and composition, and NaCl may be especially harmful in low‐calcium environments.

Another factor that may strongly influence NaCl toxicity for organisms embedded in their natural ecosystem is a previous history of salt exposure. Researchers have uncovered evidence of intraspecific variation in NaCl tolerance in a number of taxonomic groups including yeast (Bell & Gonzalez, 2011), amphibians (Albecker et al., 2021; Brady, 2012, 2013), fish (Spence et al., 2012), and zooplankton (Arnott et al., 2023; Latta et al., 2012; Liu & Steiner, 2017; Loureiro et al., 2012; Venâncio et al., 2018). In fact, a recent study demonstrated that intraspecific variation in the chloride tolerance of zooplankton species was so high that community level responses to NaCl exposure could not be predicted based on established species level toxicity thresholds (Arnott et al., 2023). Experimental evolution work with *Daphnia* demonstrated that laboratory exposure to elevated NaCl can select for increased NaCl tolerance in as few as 5–10 generations (Coldsnow, Mattes, et al., 2017). In addition, several field studies show evidence of local adaptation to elevated salinity in freshwater organisms. Early work in coastal ponds where sea salt spray created strong microgeographic salinity gradients showed that *Daphnia* genotypes dominating high‐conductivity environments were much better at withstanding acute NaCl stress (Weider & Hebert, 1987). In addition, treefrogs (*Hyla cinerea*) (Albecker et al., 2021) and the cladoceran *Simocephalus vetulus* (Loureiro et al., 2012) inhabiting coastal ponds showed evidence of local adaptation to salt stress. Spotted salamanders (*Ambystoma maculatum*) from roadside ponds showed a markedly higher tolerance of road salt (NaCl) compared with those from woodland ponds (Brady, 2012). However, wood frog (*Rana sylvatica*) populations found in the same landscape showed the reverse pattern; those from roadside ponds showed poorer fitness overall, especially in the presence of NaCl (Brady, 2013; Forgione & Brady, 2022). While the mechanisms behind this pattern of local maladaptation to road salt exposure are unclear, this finding supports that evolutionary dynamics in response to pollution stress may diverge from expected outcomes (Rogalski, 2017). Overall, this body of work shows that exposure to both sea salt and road salt (NaCl) can result in evolved increases in NaCl tolerance, although the degree to which populations change in their responses to salt exposure and even whether the response is adaptive vs. maladaptive may be difficult to predict without empirical testing.

The experimental design of toxicity trials may strongly shape observations of variation in NaCl tolerance within or among populations. Toxicity assays used in risk assessment aim to maximize reproducibility by maintaining consistent exposure conditions. For instance, nearly all toxicity assays use synthetic lake water as the test medium (Celis‐Salgado et al., 2008; Kilham et al., 1998; Klüttgen et al., 1994; Samel et al., 1999). However, if test organisms from natural populations are adapted to local lake water chemistry, the degree to which the test water solution resembles their natal lake water environment may strongly influence toxicity responses. Even for the zooplankton *Daphnia*, a model organism used in tens of thousands of toxicity trials over the past 40 years (ECOTOX Knowledgebase, n.d.), few researchers have explored the degree to which populations may be adapted to the chemistry of their lake water environment. This research supports that *Daphnia* populations can adapt to the stress of low pH (Culver & Acosta, 2018) and elevated salinity (Weider & Hebert, 1987), and show within population variation in response to calcium limitation (Alstad, 2002; Overhill, 2017): these studies manipulated single aspects of the chemical environment and used artificial lake water as the test medium. Three additional studies have explored *Daphnia* adaptation to the whole lake water environment: two used coarsely filtered lake water (allowing phytoplankton to pass through the filter) (Allen et al., 2010; Declerck et al., 2001) and one using more finely filtered lake water (Rogalski & Ferah, 2023). All three studies using natural lake water found interactions between the population of origin and the lake water environment shaping *Daphnia* life history, in some cases showing evidence of local adaptation. To date, no study has examined the extent to which adaptation to the lake water environment may influence toxicity. Our study addresses this important knowledge gap.

We conducted a set of experiments to explore how natural *Daphnia* populations respond to NaCl stress. We used filtered water from three lakes with widely divergent ion chemistry as our test medium to determine if past laboratory tests manipulating overall ionic charge and calcium availability successfully predicted NaCl toxicity in natural lake water. We incorporated a laboratory reciprocal transplant design in these toxicity trials to quantify intraspecific variation in NaCl tolerance among the populations and to determine whether toxicity increased with exposure to NaCl to a foreign lake water environment.

## 2 MATERIALS AND METHODS

### 2.1 Study system

**FIGURE 1:**
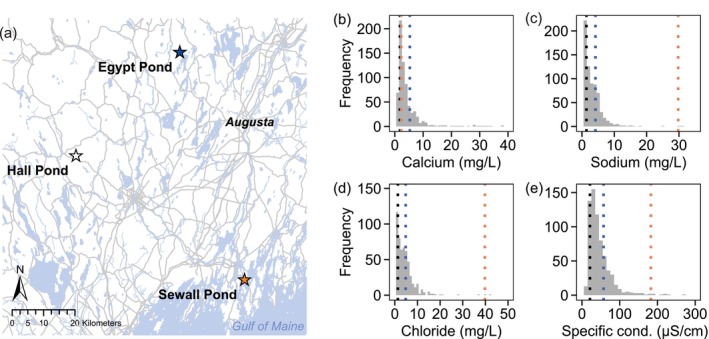
Map showing the study lakes in central and mid‐coast Maine and their ion chemistry in relation to other lakes sampled in Maine. (a) Study lake locations highlighted with stars, other waterbodies (blue), and major roadways (grey). Variation in (b) aqueous calcium (Ca^2+^), (c) sodium (Na^+^), (d) chloride (Cl^−^), and (e) specific conductance for 688 lakes sampled by the Maine Department of Environmental Protection (DEP) between 1996 and 2012 (unpublished data). Mean historic values for study lakes are marked with dotted lines (black: Hall Pond, blue: Egypt Pond, orange: Sewall Pond). Note the increase in ion availability in Sewall Pond since this sampling period (Tables 1 and 2).

In our study region of Maine, USA, lakes tend to be relatively ion‐poor compared with other freshwaters in the United States, owing to erosion resistant bedrock and sandy soils (National Lakes Assessment, 2007; Norton et al., 1989; Rogalski & Ferah, 2023). Ion availability and the relative concentrations of major cations are shaped by bedrock and soil properties, hydrology, watershed activities, and proximity to the coast (Norton et al., 1989). Our chosen study lakes represent some of the breadth of the variation in overall ion concentrations as well as calcium availability in the region (Figure 1) and support populations of our focal species, *Daphnia ambigua*. Ion‐poor Hall Pond has a specific conductance of approximately 20 μS/cm and aqueous calcium (Ca^2+^) levels of 1.3–1.6 mg/L, placing it around the 15^h^ percentile of nearly 688 lakes sampled by the Maine Department of Environmental Protection (Figure 1). Ion‐rich Sewall Pond, a lake with a specific conductance in the 99th percentile, likely receives salt inputs from atmospheric deposition (Norton et al., 1989) in addition to periodic sea salt inputs from its outlet, a brackish tidal creek (personal observation). Like other coastal Maine lakes, Sewall Pond has relatively high sodium levels (99th percentile) and moderate Ca^2+^ levels (59th percentile) given its elevated specific conductance (Rogalski & Ferah, 2023). Our third study lake, Egypt Pond, has Ca^2+^ levels far exceeding those of the other study lakes (87th percentile compared with Hall Pond's 15th percentile), with intermediate ion availability overall (50 μS/cm, placing it in the 68th percentile) (Figure 1, Tables 1 and 2).

**TABLE 1:** Chemical and physical characteristics of the three study lakes.

Lake	Year	Season	DOC	TN	TP	Secchi	pH	Cond.	SA	Und. WS	*Z*_max_
Hall	2019	Spring	–	–	–	6.60	7.17	18.20	20.6	97.5%	7.9
		Summer	–	0.23 (0.03)	10.85 (3.33)	5.90	7.18	21.30			
	2020	Fall	–	0.29	9.95	–	–	–			
	2021	Spring	–	0.24 (0.04)	5.72 (1.59)	–	–	–			
		Summer	3.35 (0.09)	0.26 (0.00)	7.71 (0.28)	5.60	–	20			
		Fall	–	0.33 (0.02)	6.62 (0.04)	–	–	–			
Egypt	2019	Spring	–	0.25	7.32	3.48 (0.33)	7.40 (0.02)	43.20 (2.40)	28.7	93.0%	15.25
		Summer	–	0.21 (0.00)	6.53 (0.54)	4.50	7.29	55.10			
	2020	Fall	–	0.29	8.83	–	–	–			
	2021	Spring	–	0.25 (0.00)	6.86 (1.15)	4.15	–	50			
		Summer	4.14 (0.08)	0.26 (0.00)	8.78 (1.76)	4.15	–	50			
		Fall	–	–	–	–	7.25	50			
Sewall	2019	Spring	–	0.26	39.14	1.73 (0.13)	6.13 (0.15)	421.2 (13.8)	17.4	92.3%	3.7
		Summer	–	0.32 (0.08)	17.41 (9.30)	1.23 (0.03)	6.69 (0.06)	410.1 (25.0)			
		Fall	–	–	–	1.20	6.67	502.0			
	2020	Spring	–	–	–	–	–	265.7			
		Fall	–	–	–	–	–	455.2			
	2021	Spring	–	0.32 (0.01)	9.10 (1.84)	–	–	–			
		Summer	5.14 (0.04)	0.27 (0.00)	13.57 (0.34)	2.21	–	420			
		Fall	–	0.65 (0.02)	15.10 (2.51)	0.75	6.85	460			

*Note*: Total nitrogen (TN) and total phosphorus (TP) measurements of unfiltered lake water samples were collected at 1 m depth. Trial 1 (Hall and Egypt) and trial 2 (Hall and Sewall) took place in the summer of 2020 and 2021, respectively. Dissolved organic carbon (DOC) was measured for each study lake in June ‘21 and includes lake water used in trial 2 (Hall and Sewall). pH and specific conductance (Cond.) represent in situ surface field measurements with a YSI‐ProDSS probe or Oakton EcoTstr (specific conductance measured in 2021). DOC and TN concentrations in mg/L. TP in μg/L. Specific conductance is in μS/cm. Lake surface area (SA) in hectares. The secchi depth and lake maximum depth (*Z*_max_) in m. Percent of the watershed consisting of undeveloped cover (Und. WS) based on analysis of satellite imagery in ArcGIS.

**TABLE 2:** Concentrations of major ions in filtered lake water from the three study lakes used in trials 1 (‘20) and 2 (‘21).

Trial	Lake	Treatment	Na^+^	K^+^	Mg^2+^	Ca^2+^	SO_4_^2−^	Cl^−^	Added Na^+^	Added Cl^−^
2020	Hall	Control	1.07 (0.15)	0.23 (0.01)	0.28 (0.01)	1.71 (0.02)	0.55 (0.04)	4.08 (0.03)		
		NaCl	341.6 (1.31)	0.23 (0.01)	0.27 (0.00)	1.78 (0.00)	0.64 (0.00)	507.9 (1.22)	340.5	503.8
	Egypt	Control	2.17 (−)	0.44 (−)	0.61 (−)	7.12 (−)	1.02 (−)	5.78 (−)		
		NaCl	339.7 (0.50)	0.40 (0.01)	0.57 (0.00)	7.01 (0.08)	1.04 (0.00)	507.9 (0.56)	337.5	502.1
2021	Hall	Control	1.72 (0.32)	0.17 (0.03)	0.24 (0.05)	1.28 (0.04)	0.62 (0.01)	4.19 (0.04)		
		NaCl	321.3 (7.37)	0.20 (0.07)	0.18 (0.06)	1.24 (0.05)	0.63 (0.01)	473.0 (6.59)	319.6	468.9
	Sewall	Control	60.47 (0.36)	2.42 (0.01)	6.69 (0.09)	3.36 (0.08)	4.87 (0.05)	105.5 (0.73)		
		NaCl	363.1 (11.90)	2.20 (0.03)	6.37 (0.07)	3.12 (0.08)	4.82 (0.06)	577.7 (16.0)	302.6	472.1

*Note*: Mean values for major cations and anions (mg/L) in control and NaCl spiked treatments are provided, with standard errors in parentheses. Due to sample damage during shipping, only one control sample from Egypt Pond was measured. Major ions were analyzed with a Dionex Ion Chromatograph ICS‐1100 (Thermo Fischer Corporation).

Field sampling in 2019–2021 indicates that Hall Pond and Egypt Pond are oligotrophic, with total phosphorus (TP) levels typically below 10 μg/L (Table 1). Sewall Pond varies between mesotrophic to eutrophic, though total phosphorus levels in summer ‘21, when our trial was conducted, were more modest (9.10 μg/L). Sewall Pond has higher levels of dissolved organic carbon and a lower pH compared with the other two study lakes (Table 1). All three lakes have experienced little development within their watersheds. Sewall Pond's watershed is the most developed with 5.6% low‐density residential development and 2% state road. Egypt Pond's watershed includes a local road (1.4%) and pastureland (5.6%); Hall Pond's watershed includes a gravel road (1.3%) and low‐density residential cover (1.2%).

Further details on the water chemistry of the study lakes relative to other coastal and inland Maine lakes are available in Rogalski and Ferah (2023).

### 2.2 *Daphnia* isolation and culturing conditions

*Daphnia* life history in temperate latitudes typically involves an asexual, parthenogenetic period during the growing season (e.g., spring‐fall) and sexual production of dormant embryos to escape stress (e.g., winter, intense parasitism or predation pressure) (Gyllström & Hansson, 2004; Thielsch et al., 2009). Thus, individuals observed in the water column in late spring are likely clonal replicates of those individuals that hatched from the resting egg bank produced by past generations of *Daphnia*.

In late‐May of 2019, we established isofemale clonal lineages of *Daphnia ambigua* from each of our study lakes from net collections (80 μm mesh). We sampled the full water column from the deep basin of each lake, kept the sample in unfiltered lake water in cool dark conditions, and then returned to the lab and processed the samples within 2 h. We isolated all *D. ambigua* individuals from random subsamples until at least 12 animals were collected from each lake. We maintained each *Daphnia* clonal lineage asexually in filtered water from their respective lake for two generations (about 28 days) and then randomly selected four lineages to maintain for further study. For more information see Rogalski and Ferah (2023).

Prior to our experiments, which took place in the summers of 2020 and 2021, we maintained the *Daphnia* clonal lineages from each of the three study lakes for 25+ generations. We kept *Daphnia* individually in borosilicate beakers with 25 mL filtered water (Pall A/E, 1.0 μm pore size) from their respective lake of origin. The key purpose of our trials was to examine the influence of variation in natural lake water chemistry and potential adaptation to that lake water environment in shaping NaCl toxicity. Thus, aside from the lake water environment, we kept other environmental conditions consistent among *Daphnia* populations, including those that may influence toxicity responses such as food availability or quality (Brown & Yan, 2015).

*Daphnia* cultures were housed in incubators (Percival I‐41 VL) at 20°C with a 16‐h light: 8‐h dark period. We changed the lake water and removed offspring twice weekly and fed each *Daphnia* 500,000 cells of *Ankistrodesmus falcatus* four times weekly. *Ankistrodesmus* was cultured in heat sterilized modified ASM‐1 medium (Goulden & Hornig, 1980) at room temperature, harvested weekly during the logistic growth phase, and stored at 4°C. A vitamin mixture (Goulden et al., 1982) was added to the algal culture to support *Daphnia* nutritional needs. After harvesting, algae were settled and resuspended with filtered lake water, maintaining a density of 1,000,000 cells/mL using a hemocytometer. *Daphnia* were fed resuspended algae mixed with the same lake water used for their culturing. Further details on culturing conditions are available in (Rogalski & Ferah, 2023).

### 2.3 Experimental design

We conducted a laboratory reciprocal transplant experiment including both control and NaCl spiked treatments, in both the home and transplant lake water, to examine the potential interactions between lake water chemistry and local adaptation to the lake water environment in shaping intraspecific variation in NaCl toxicity. We performed this experiment in two trials. The first trial paired *Daphnia* from low‐Ca^2+^/ low‐Cl^−^ Hall Pond and high‐Ca^2+^/ low‐Cl^−^ Egypt Pond. The second trial paired *Daphnia* from low‐Ca^2+^/ low‐Cl^−^ Hall Pond and mid‐Ca^2+^/ high‐Cl^−^Sewall Pond. Using 3–4 *Daphnia* clonal lineages from each population (one of four lineages was lost from Sewall Pond), we assessed survival time, offspring production, and intrinsic rate of growth (*r*) of asexually produced replicates cultured in filtered lake water from either their home or transplant lake water, with or without added stress of 825 mg/L NaCl (reagent grade, Fisher Scientific). This NaCl concentration represents approximately 25–50% of the 48‐h LC_50_ value estimated in pilot experiments exposing *Daphnia* from each population to NaCl in each of the lake water environments. We expected this concentration to be high enough to cause chronic toxicity in the least stressful treatment but low enough to avoid complete mortality in the most sensitive treatment, based on published acute‐to‐chronic NaCl toxicity ratios (Benoit & Stephan, 1988).

Maternal provisioning has been shown to be an important source of calcium for *Daphnia* neonates, particularly during early development (Giardini et al., 2015). In addition, *Daphnia* offspring produced by mothers that have experienced moderately elevated salinity conditions may show increased NaCl tolerance in the F1 (but not the F2) generation (Venâncio et al., 2018). For these reasons, immediately preceding the trials, we reared *Daphnia* in their control test water conditions for one generation (14–20 days), by transferring *Daphnia* neonates to filtered lake water from either their home lake or the paired comparison lake. We initiated the trials in the next generation, using third to fifth brood *Daphnia* neonates aged 6–24 h from each clonal lineage, transferred to the same type of filtered lake water used in their acclimation generation with or without 825 mg/L added NaCl. In this way, we aimed to minimize maternal effects in response to the control test water conditions while evaluating genetic variation in tolerance for NaCl in that environment. We included 10 replicate *Daphnia* individuals per lake (2) × clonal lineage (3–4) × lake water (2) × NaCl treatment (2) in the trials (Trial 1: *N* = 320; Trial 2: *N* = 280).

We checked for mortality and counted and removed any offspring produced daily. On day 14 we terminated the trials. We calculated intrinsic rates of increase (*r*) from life‐table data using the Euler‐Lotka equation (Stearns, 1992),

$$1=\sum_{x=0}^{k}e^{-\mathrm{rx}}l_{x}m_{x},$$

where *l*_*x*_ is the proportion of individuals surviving to day *x* and *m*_*x*_ is the mean number of offspring produced per surviving *Daphnia* on day *x*. We calculated *r* iteratively for each clonal lineage × lake water × NaCl treatment combination (Trial 1: *N* = 32; Trial 2: *N* = 28). Three Hall Pond clones and one Egypt Pond clone failed to reproduce in Hall Pond water with added NaCl. To calculate *r* in these cases, we designated a value of 0.01 for offspring produced on day 14, which yielded an *r* of −0.3289.

### 2.4 Statistical analyses

We used generalized linear mixed models (GLMMs) to evaluate the extent to which the lake water source (Environment: E), population of origin (Population: P), NaCl treatment (control: no NaCl added; NaCl: 825 mg/L NaCl added) and any interactions among these variables (P × E × NaCl treatment) explained patterns in survival duration, offspring production, or *r*. We evaluated data from the two trials (trial 1: low‐Ca^2+^/low‐Cl^−^ Hall Pond vs. high‐Ca^2+^/low‐Cl^−^ Egypt Pond; trial 2: low‐Ca^2+^/low‐Cl^−^ Hall Pond vs. mid‐Ca^2+^/high‐Cl^−^ Sewall Pond) separately.

We examined survival patterns as survival time, in days (Gaussian distribution). There were many individuals that failed to reproduce in the trial, particularly in the NaCl treatment. To account for the resulting overdispersion, we examined reproduction using a hurdle model, first examining whether an animal reproduced during the 14‐day trial (binomial response variable) and then the total number of offspring produced for those individuals that reproduced (zero truncated Poisson distribution). In addition, we examined the timing of first reproduction (Gaussian distribution) for those *Daphnia* that reproduced.

We selected the best fit model for each response variable by comparing the Akaike information criterion (AIC) of the saturated model with nested simpler models containing fewer fixed effects. We used likelihood ratio tests to select which interactions among the fixed effects were warranted for inclusion in the final model, based on Zuur et al. (2009). We included clonal lineage as a random intercept. We evaluated the normality of the residuals of the selected models using residual versus fitted plots and normal quantile‐quantile plots.

All analyses were conducted using the statistical program R (v. 4.2.1) (R Core Team, 2016). We used the “glmmTMB” package (Brooks et al., 2017) for mixed effects modeling for all response variables except the likelihood of reproduction, which was complicated by the complete separation of the data (100% reproduction was observed in some combinations of P × E × NaCl treatment groups). For these analyses we fitted a Bayesian GLMM using the blme package (Dorie et al., 2021), specifying weakly informative priors as recommended by Chung et al. (2013). Differences in survival, reproduction, and *r* among all pairwise comparisons were evaluated using post‐hoc tests of Tukey's Honest Significant Difference (HSD) with the “emmeans” package.

### 2.5 Predictions

We predicted that the population of origin, lake water environment, and NaCl treatment would interact to shape NaCl toxicity (Figure S1). We expected elevated calcium in Egypt Pond's water to mitigate NaCl toxicity compared with the relatively from low‐Ca^2+^ conditions in Hall Pond (Mount et al., 2016) (significant water × treatment interaction, Figure S1). In addition, we expected *Daphnia* from Sewall Pond to be more tolerant of added NaCl stress (significant population × treatment interaction, Figure S1) since NaCl concentrations in Sewall Pond are about 20 times greater than levels in both lower‐ion lakes (Table 2). Last, we predicted that *Daphnia* would show evidence of local adaptation to the lake water environment, performing relatively better in their natal lake water environment relative to a population from a foreign environment (significant origin × lake water interaction, Figure S1).

## 3 RESULTS

### 3.1 Trial 1: Hall Pond (low‐Ca^**2**^ ^+^/low‐Cl ^−^) versus Egypt Pond (high‐Ca^**2**^ ^+^/low‐Cl ^−^)

#### 3.1.1 Survival

**FIGURE 2:**
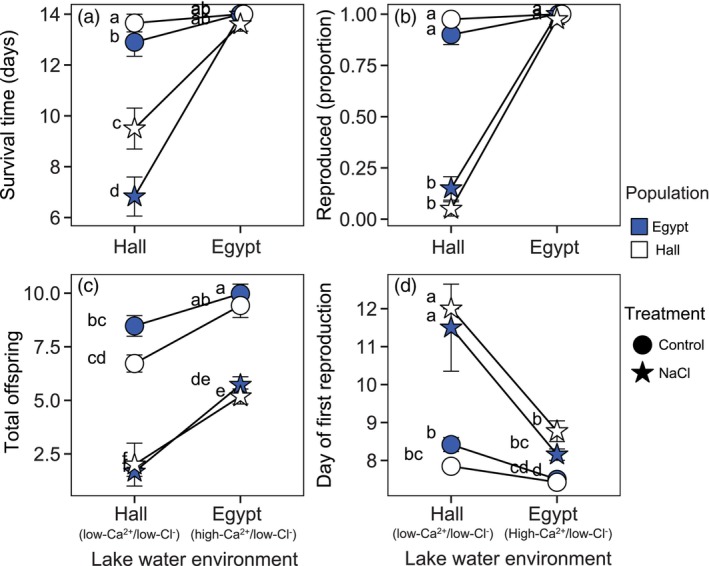
*Daphnia* survival and reproduction were shaped by interactions among the lake water environment (x‐axis), NaCl treatment, and/or population of origin in trial 1, pairing low‐Ca^2+^/ low‐Cl^−^ Hall Pond and high‐Ca^2+^/low‐Cl^−^ Egypt Pond. Mean values for survival duration (a), the likelihood of reproduction (b), total offspring produced by individuals that reproduced (c), and timing of first reproduction (d) include replicates from all clonal lineages from Hall Pond (white) or Egypt Pond (blue), in either control (circles) or NaCl spiked (star) conditions. Error bars show ±1 standard error (SE) of the mean across clonal replicates (*N* = 40 for each population and treatment). Data points sharing the same letter (a–e) showed no significant difference (*p* > 0.05) in post‐hoc Tukey tests of GLMM results. A slight horizontal jitter was introduced to display overlapping data points.

Survival in the Hall Pond (low‐Ca^2+^/low‐Cl^−^) versus Egypt Pond (high‐Ca^2+^/low‐Cl^−^) trial was strongly shaped by the interaction of lake water and NaCl treatment (*p* < 0.0001, *χ* ^2^ = 49.69, ΔAIC = −47.7). Survival was unimpacted by the addition of NaCl in Egypt Pond's high‐ Ca^2+^ lake water (Tukey HSD, *p* = 0.969, *t* ratio = 0.45, Figure 2a), but NaCl reduced survival time by about 5 days on average in Hall Pond's low‐Ca^2+^ lake water (Tukey HSD, *p* < 0.0001, *t* ratio = 10.89). In addition, the interaction between population and lake water environment improved the model fit (*p* = 0.004, *χ* ^2^ = 8.20, ΔAIC = −6.2). *Daphnia* originating from Egypt Pond showed lower survival rates in Hall Pond's low‐Ca^2+^ lake water, in both the control and NaCl treatments (Tukey HSD, *p* = 0.007, *t* ratio = −3.65).

#### 3.1.2 Reproduction

The effects of NaCl on reproduction were greatly reduced in Egypt Pond's high‐Ca^2+^/low‐Cl^−^ water. The best fit model for predicting whether *Daphnia* reproduced included interactions between the lake water and NaCl treatment as well as a population of origin × NaCl treatment interaction (*p* = 0.021, *χ* ^2^ = 5.3113, AIC = 108.74). In Egypt Pond's water, most *Daphnia* individuals produced offspring whether in the control or NaCl treatment (98% + reproduced), while the ability to reproduce was strongly suppressed with NaCl added to Hall Pond's ion‐poor environment, whether *Daphnia* originated from Hall or Egypt Pond (Figure 2b; Tukey HSD for Hall *Daphnia* in Hall water: *p* < 0.0001, *z* ratio = 5.99; for Egypt *Daphnia* in Hall water: *p* < 0.0001, *z* ratio = 5.80). For those *Daphnia* that produced offspring, fecundity was also explained by a lake water × NaCl treatment interaction (*p* < 0.0001, *χ* ^2^ = 18.803, ΔAIC = −16.8). Over the 14‐day trial, NaCl reduced total fecundity by about 85% in Hall Pond's water compared with a 44% decrease in Egypt Pond's water (Figure 2c). In addition, *Daphnia* from both populations experienced a sizeable 27.5% reduction in fecundity in the control treatment in Hall Pond water (Tukey HSD *p* < 0.001, *t* ratio = −4.44). Variation in the timing of first reproduction was explained by interactions between lake water and NaCl treatment (*p* < 0.0001, *χ* ^2^ = 30.89, ΔAIC = −28.9) and between the population of origin and NaCl treatment and (*p* = 0.001, *χ* ^2^ = 10.20, ΔAIC = −8.2). NaCl caused a 3.65‐day delay in the onset of reproduction in Hall Pond's water (Figure 2d; Tukey HSD *p* < 0.0001, *t* ratio = −9.10), but only a 1‐day delay in Egypt Pond's high‐Ca^2+^ water (Tukey HSD, *p* < 0.0001, *t* ratio = −5.89). *Daphnia* from Hall Pond were slightly more strongly impacted by NaCl compared with those from Egypt Pond (Figure 2d).

#### 3.1.3 Intrinsic rate of increase (*r*)

**FIGURE 3:**
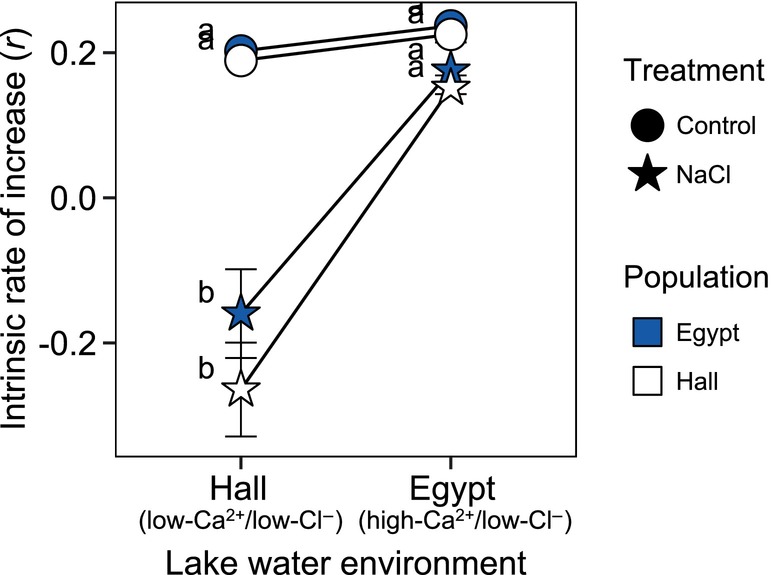
The lake water environment and NaCl treatment interacted to shape the intrinsic rate of increase (*r*) for *Daphnia* in trial 1. Mean *r* values (±1SE) for clonal lineages from Hall Pond (white) and Egypt Pond (blue) when reared under control (circles) or NaCl spiked (stars) conditions in each lake water environment (x‐axis). Data points sharing the same letter (a‐b) showed no significant difference (*p* > 0.05) in post‐hoc Tukey tests of GLMM results.

We observed a significant lake water environment × NaCl treatment interaction in predicting *r* in the first trial (*p* < 0.0001, *χ* ^2^ = 36.41, ΔAIC = −34.41). While NaCl had no effect on *r* in Egypt Pond's mid‐ion/high‐Ca^2+^ water for either population (Tukey HSD: *p* = 0.349, *t* ratio = 2.26), *r* shifted from rapid exponential growth to rapid population decline with the addition of NaCl in Hall Pond's low‐Ca^2+^/low‐Cl^−^ environment (Tukey HSD: *p* < 0.0001, *t* ratio = 13.59) (Figure 3). There was no significant effect of population of origin on *r* (*p* = 0.129, *χ* ^2^ = 2.30, ΔAIC = −0.28), however, we included this variable in the final model to account for a lack of independence in the data.

### 3.2 Trial 2: Hall Pond (low‐Ca^2^ ^+^/low‐Cl ^−^) vs. Sewall Pond (mid‐Ca^2^ ^+^/high‐Cl ^−^)

#### 3.2.1 Survival

**FIGURE 4:**
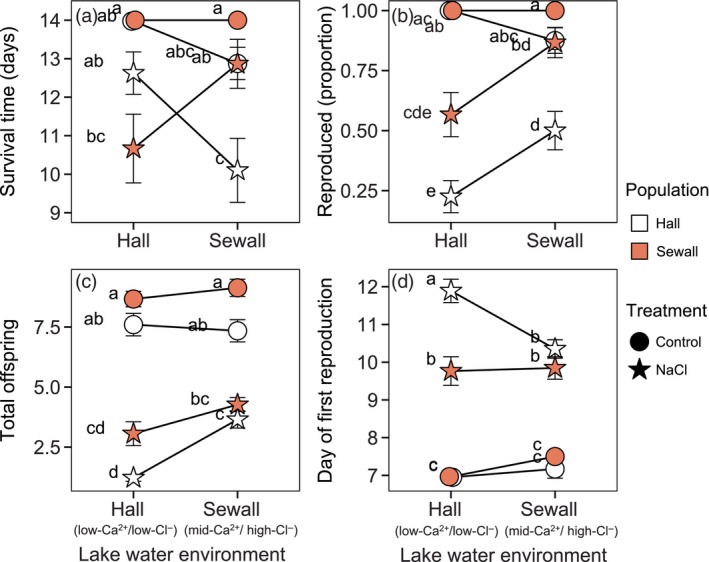
The lake water environment (x‐axis), population of origin, and NaCl treatment interacted to shape survival time and reproduction in the second trial, pairing low‐Ca^2+^/low‐Cl^−^ Hall Pond and mid‐Ca^2+^/high‐Cl^−^ Sewall Pond. Mean values for survival duration (a), the likelihood of reproduction (b), total offspring produced by individuals that reproduced (c), and timing of first reproduction (d) include replicates from all clonal lineages from Hall Pond (white) or Egypt Pond (blue), in either control (circles) or NaCl spiked (star) conditions. Error bars represent ±1 SE of the mean (*N* = 30–40 for each population and treatment). Data points sharing the same letter (a–e) showed no significant difference (*p* > 0.05) in post‐hoc Tukey tests of GLMM results. A slight horizontal jitter was introduced to display overlapping data points.

In the second trial, the best fit model for survival time included the full three‐way interaction among the population of origin, lake water environment, and NaCl treatment (*p* = 0.014, *χ* ^2^ = 6.021, ΔAIC = −4.0). For *Daphnia* from both populations, NaCl toxicity impacted survival much more strongly in the foreign lake water environment (Figure 4a). In their home lake water environments, NaCl reduced survival by a little over one day, but the effect was statistically indistinguishable from the control treatment (Tukey HSD for Hall *Daphnia* in Hall Pond water: *p* = 0.509, *t* ratio = 1.96; for Sewall *Daphnia* in Sewall Pond water: *p* = 0.844, *t* ratio = 1.43). In the foreign lake water environments, added NaCl reduced survival by about 3 days for *Daphnia* from both populations (Tukey HSD for Hall *Daphnia* in Sewall water: 2.78‐day reduction in survival, *p* = 0.002, *t* ratio = 4.04; for Sewall *Daphnia* in Hall water: 3.33‐day reduction in survival, *p* < 0.001, *t* ratio = 4.20).

#### 3.2.2 Reproduction

We observed an interaction between lake water and NaCl treatment in predicting whether *Daphnia* reproduced in the second trial, where the likelihood of reproduction greatly decreased with NaCl addition in Hall Pond's low‐Ca^2+^/low‐Cl^−^ lake water (*p* < 0.001, *χ* ^2^ = 12.505, ΔAIC = −10.5, Figure 4b). In addition, *Daphnia* from Hall Pond were less likely to reproduce overall than those from mid‐Ca^2+^/high‐Cl^−^ Sewall Pond (fixed effect of population of origin: *p* = 0.024, *χ* ^2^ = 5.06, ΔAIC = −3.06). *Daphnia* from ion‐poor Hall Pond appear to be more strongly impacted by NaCl in both lake water environments, relative to Sewall Pond *Daphnia*, but the interaction between treatment and population of origin was not significant (*p* = 0.462, *χ* ^2^ = 0.54, ΔAIC = +1.46). For those *Daphnia* that reproduced, the best fit model predicting total fecundity included a three‐way interaction among the population of origin, lake water, and NaCl treatment (*p* < 0.001, *χ* ^2^ = 11.03, ΔAIC = −9.03). NaCl led to a sizeable decrease in fecundity, especially in Hall Pond's water (Figure 4c); Hall Pond *Daphnia* experienced an even greater decline in offspring production in this stressful environment (96% reduction vs. 68% for Sewall Pond *Daphnia*). The best fit model for the timing of first reproduction also included a three‐way interaction among the population of origin, lake water environment, and NaCl treatment (*p* = 0.032, *χ* ^2^ = 4.63, ΔAIC = −2.63). For those individuals that reproduced, the first brood was delayed by about three days in the NaCl treatment; however, Hall Pond *Daphnia* reared in Hall Pond's low‐ion water experienced a 5‐day delay with added NaCl (Figure 4d).

#### 3.2.3 Intrinsic rate of increase (*r*)

**FIGURE 5:**
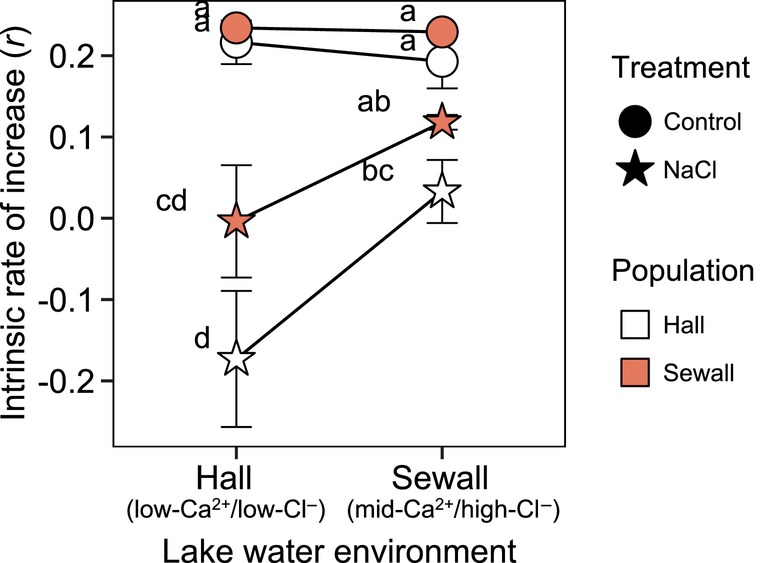
Interactions among the lake water environment, population of origin, and NaCl treatment shaped the intrinsic rate of increase (*r*) for *Daphnia* in trial 2. Mean *r* values (±1SE) for clonal lineages from Hall Pond (white) and Egypt Pond (blue) when reared under control (circles) or NaCl spiked (stars) conditions in each lake water environment (x‐axis). Data points sharing the same letter (a–d) showed no significant difference (*p* > 0.05) in post‐hoc Tukey tests of GLMM results.

The best fit model that predicted variation in *r* in the second trial included interactions between the lake water environment and NaCl treatment (*p* < 0.001, *χ* ^2^ = 12.64, ΔAIC = −10.64) as well as the population of origin and treatment (*p* = 0.017, *χ* ^2^ = 5.65, ΔAIC = −3.65). *Daphnia* from Sewall Pond showed a modest, non‐significant decrease in *r* in their natal lake water with added NaCl (Tukey HSD: *p* = 0.489, *t* ratio = 2.07), while NaCl exposure in low‐ion Hall Pond water led to a net‐zero growth rate (Tukey HSD: *p* < 0.001, *t* ratio = 6.77). *Daphnia* originating from Hall Pond also experienced a greater decrease in *r* with NaCl exposure in their natal lake water compared with exposure in Sewall Pond water; however, the effect of NaCl on Hall Pond *Daphnia* in both lake water environments was about double that experienced by Sewall Pond *Daphnia* (Figure 5).

## 4 DISCUSSION

Chronic exposure to toxic levels of NaCl led to reduced survival duration, delayed reproduction, increased sterility, reduced fecundity, and reduced intrinsic rate of increase (*r*) in our laboratory reciprocal transplant toxicity trials. However, the degree to which *Daphnia* were impacted by NaCl toxicity relied strongly on their population of origin and the lake water environment in which they were reared. One striking result was the great reduction in NaCl toxicity for *Daphnia* when reared in Egypt Pond's high‐Ca^2+^/low‐Cl^−^ lake water. While 825 mg/L of added NaCl led to dramatically reduced survival and a near cessation of reproduction in Hall Pond's low‐Ca^2+^ water, these effects of NaCl in Egypt Pond's high‐Ca^2+^ water were modest or even absent. Significant interactions between the population of origin (P) and lake water environment (E) in shaping survival in both trials were consistent with the prediction that adaptation to natal lake water conditions may impact NaCl toxicity. This pattern was most evident in trial 2, pairing ion‐rich Sewall Pond and ion‐poor Hall Pond, where the negative impact of NaCl on survival time was 2–3 times greater in the foreign lake water environment for both populations. However, we did not observe this P × E interaction with respect to reproduction or overall fitness (*r*) in either trial, suggesting that the survival patterns we observed may be more an indication of life history trade‐offs than adaptation to the test water environment. Lastly, *Daphnia* from Sewall Pond were the most tolerant of NaCl exposure, indicating that their ion‐rich natal environment selected for increased NaCl tolerance. In both test water environments, Sewall Pond *Daphnia* were better able to reproduce and showed greater overall fitness (*r*) under NaCl spiked conditions. Altogether, these findings support that lake water chemistry can strongly influence *Daphnia* responses to NaCl pollution, both directly by influencing toxicity and more indirectly by driving population‐level differences in NaCl tolerance and life history responses.

**FIGURE 6:**
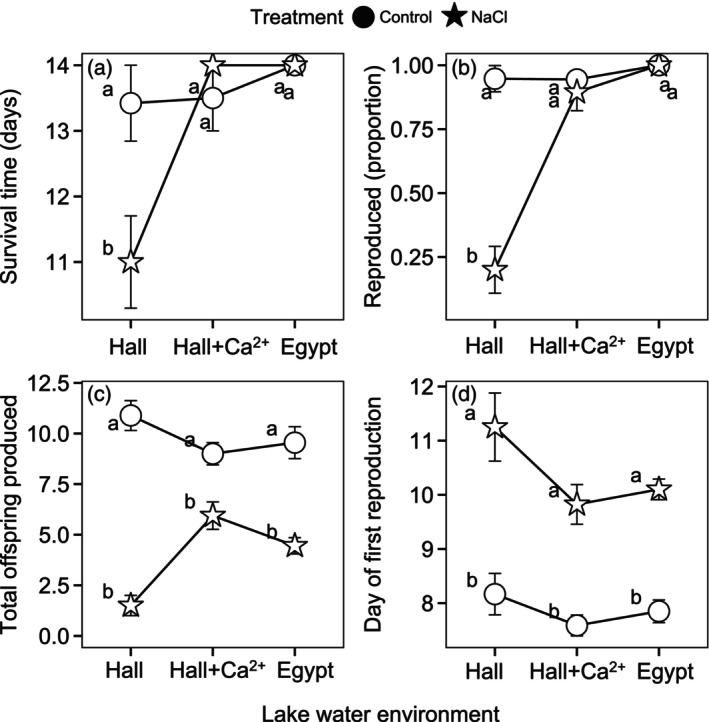
Impacts of added NaCl on the performance of Hall Pond *Daphnia* reared in lake water from their natal lake, Egypt Pond, and calcium‐supplemented Hall Pond water. Survival duration (a), the likelihood of reproduction (b), total offspring produced during the 14‐day trial (c), and timing of first reproduction (d) show mean values of replicates across both clonal lineages (±1SE) in one of three test media (x‐axis): low‐Ca^2+^/low‐Cl^−^ Hall Pond water, high‐Ca^2+^/low‐Cl^−^ Egypt Pond water, and Hall Pond water with calcium added to equal Egypt Pond concentrations (Table S1). Circles show control treatments and stars NaCl conditions. a, b designates pairwise differences in post‐hoc Tukey tests of GLMM results, where the same letter indicates no significant difference (*p* > 0.05). See Supplementary text S1 for additional details on this trial.

The most persistent pattern observed in our study was the strong impact of the lake water environment on NaCl toxicity, as indicated by a significant lake water × NaCl treatment interaction in predicting nearly every measure of survival and reproduction in both trials. NaCl was relatively more toxic in Hall Pond's low‐Ca^2+^/low‐Cl^−^ environment compared with test waters from the other two study lakes. Calcium availability is the likely driver behind the substantial decrease in NaCl toxicity observed in Egypt Pond's lake water environment. Calcium levels were about 4 × higher in Egypt Pond compared with Hall Pond when the experiment was conducted, while other cations were only about twice as high (Table 2). In addition, a subsequent trial experimentally adding calcium to Hall Pond water to match concentrations in Egypt Pond showed a substantial decrease in NaCl toxicity, closely replicating NaCl impacts in water from Egypt Pond (Figure 6, Supplementary Text S1). This finding is consistent with past research demonstrating decreasing NaCl toxicity with increasing calcium availability (Elphick et al., 2011; Mount et al., 2016; Soucek et al., 2011). In one study, chronic NaCl tolerance in *Ceriodaphnia dubia* increased log‐linearly as calcium concentrations increased from 4 to 64 mg/L (Elphick et al., 2011). Our observation of substantially reduced NaCl toxicity for *Daphnia ambigua* reared in lake water with only 7.1 mg/L Ca^2+^ suggests that even relatively subtle variation in calcium availability may strongly influence road salt toxicity in landscapes supporting ion‐poor freshwater environments.

Identifying a single driver of decreased NaCl toxicity in Sewall Pond's lake water is difficult because the water chemistry of Sewall Pond and Hall Pond differs along several axes. Moderately elevated calcium levels in Sewall Pond could explain the lower NaCl toxicity (1.6× higher in Sewall Pond). However, this modest increase in calcium in Sewall Pond's water is accompanied by much more dramatic increases in concentrations of other major ions including magnesium (6.7× higher), sulfate (6.8× higher), sodium (34× higher), and chloride (24× higher), consistent with the influence of coastal sea salt inputs on the water chemistry in Sewall Pond (Table 2). We might expect the general increase in osmolarity associated with Sewall Pond's ion‐rich environment to increase NaCl toxicity (Erickson et al., 2017), yet we observe the opposite effect, at least with respect to reproduction and growth. Researchers have observed little impact of magnesium (Mount et al., 2016) or sulfate (Mount et al., 2016; Soucek et al., 2011) on NaCl toxicity in cladocerans, though these interactions have received less attention than the effects of calcium. Other key differences between these two lakes are the pH of Sewall Pond water is about an order of magnitude more acidic than that of Hall Pond (5.98–6.55 vs. 6.83–7.02 in surface waters) and Sewall Pond has higher levels of dissolved organic carbon (DOC) (5.14 mg/L vs. 3.35 mg/L). Exploration into the effects of these water chemistry parameters on NaCl toxicity is limited and findings are mixed. Lower pH may inhibit sodium and chloride uptake, potentially reducing NaCl toxicity, but may also increase toxicity by increasing the permeability of ionocytes in gill epithelia (Griffith, 2017). Over a pH range of 6.75–8.2 Mount et al. (2016) saw no effect of pH on NaCl toxicity in *Ceriodaphnia*. Increased dissolved organic matter has been shown to reduce sodium transport in zebrafish (Al‐Reasi et al., 2016) but had no effect on Na^+^ uptake or excretion in *Daphnia* (Al‐Reasi et al., 2013). Further exploration into how ion composition, pH, dissolved organic matter, and other aspects of lake water chemistry influence NaCl toxicity will aid in predicting the extent to which similar environments may be buffered from the effects of road salt pollution.

The reciprocal transplant aspect of our experimental design revealed that while the lake water environment was an important predictor of NaCl toxicity, *Daphnia* populations varied substantially in how they responded to the various lake water and NaCl treatment combinations. In both trials, variation in survival time was explained by a significant population of origin × lake water environment interaction, such that survival duration decreased in the foreign lake water environment (Figure S1d). In the first trial, Hall Pond *Daphnia* survived longer in their natal lake water compared with *Daphnia* from Egypt Pond, both in the control and NaCl spiked environments. In the Hall vs. Sewall Pond trial, a three‐way P × E × NaCl treatment interaction indicated that the effects of NaCl on survival time were greatly diminished in the home lake water environment for both populations. These survival patterns are consistent with predictions of adaptation to the natal lake water environment influencing *Daphnia* NaCl toxicity responses, particularly the classic crossing interaction observed in the Hall vs. Sewall Pond trial (Figure 4a). Given that acute toxicity tests, focused on mortality as a key response variable, are a common currency in the regulation and mitigation of pollution impacts (ECOTOX Knowledgebase, n.d.), this pattern of *Daphnia* populations performing better in their natal lake water environment warrants further investigation.

In contrast, we observed little evidence of local adaptation to the lake water environment with respect to reproduction and *r*. The intrinsic rate of increase (*r*) serves as an integrative measure of fitness that incorporates survival duration, the timing of maturity, and age‐dependent fecundity to estimate the potential for population growth, and thus may be considered a better indicator of adaptation. In our trials, variation in *r* closely resembled patterns of fecundity (Figures 3 and 5): the negative effect of NaCl on *r* increased in Hall Pond's low‐Ca^2+^/low‐Cl^−^ environment relative to the other two lake water environments (E × NaCl treatment interaction), but we saw no evidence of local adaptation (P × E interaction) with this measure. Instead, we observed something more complex that suggests *Daphnia* from the two lower‐ion environments (Hall Pond and Egypt Pond) may experience a trade‐off between survival and reproduction in the face of NaCl stress in a foreign lake water environment. While *Daphnia* from Hall Pond experience a smaller reduction in survival with added NaCl in the Hall Pond environment, compared with *Daphnia* from Egypt Pond, the Hall Pond *Daphnia* experienced a greater reduction in reproduction (Figure 2b) and greater delay of maturity (Figure 2d). Consequently, NaCl exposure in Hall Pond water led to very similar *r* for *Daphnia* from Hall Pond and Egypt Pond (Figure 3). Hall Pond *Daphnia* displayed a similar dynamic in the second trial, where increased survival duration in the Hall Pond NaCl spiked environment is accompanied by greatly delayed maturity and more frequent sterility (Figures 4 and 5). As a result, overall fitness (*r*) in trial 2 was not improved for Hall Pond *Daphnia* exposed to NaCl in their natal lake water compared with Sewall Pond's water. Interestingly, *Daphnia* from Sewall Pond did not show evidence of such a trade‐off. Instead, *Daphnia* from this population show greater survival time, greater likelihood of reproduction, and no additional delay in the onset of reproduction in their natal lake water compared with the low‐ion Hall Pond environment. While our study is not designed to explicitly evaluate survival‐reproduction trade‐offs (Stearns, 1992), a similar pattern in differential investment in fitness components and toxicant resistance was observed in *Daphnia* in response to copper exposure (Agra et al., 2011).

After accounting for the effects of the lake water environment, the population of origin, and their interaction, *Daphnia* from ion‐rich Sewall Pond stood out as having the highest tolerance for added NaCl. Sewall Pond *Daphnia* experienced reduced impacts of NaCl on reproduction and *r* relative to Hall Pond *Daphnia*, even in the relatively stressful, ion‐poor Hall Pond environment. On average, Sewall Pond *Daphnia* were able to achieve a net zero intrinsic growth rate in Hall Pond's water with added NaCl, while those from the other two populations showed substantial negative values for *r* (Figures 3, 5). Levels of chloride in Sewall Pond water (control treatment) at the time of the trial were about 105 mg/L, similar to levels observed when the Sewall Pond clones were collected from the lake (115 mg/L). *Daphnia pulex* inhabiting high‐salinity rock bluff and tundra ponds impacted by sea spray also showed substantially elevated tolerance for NaCl (Weider & Hebert, 1987). The salinity of Sewall Pond is modest in comparison to these Canadian sea salt‐impacted habitats, but the NaCl concentrations in Sewall Pond are similar to the lowest levels shown to elicit an evolutionary response to NaCl exposure in *Daphnia pulex* (Coldsnow, Mattes, et al., 2017). Our study supports that even modest exposure to sea salt may select for increased NaCl tolerance in *Daphnia*, while populations naïve to elevated NaCl concentrations (i.e., inland lakes with undeveloped watersheds) are likely to be more sensitive to added salt stress.

## 5 CONCLUSIONS

In environmental risk assessments, typically neither environmental nor genetic variation are considered in estimating the impacts of toxicant exposure and in making guidelines for pollution mitigation and remediation. Yet a strong case has been made that ignoring the ecological and evolutionary complexity surrounding toxicology may grossly underestimate the long‐term impacts of pollution on ecosystems (Brady, Monosson, et al., 2017; Straub et al., 2020; Topping et al., 2020). Our study builds on this work by showing that environmental impacts on toxicity and intraspecific genetic variation in tolerance may interact in ways that alternately reduce (in Sewall Pond) or increase (in Hall Pond) toxicity. If we had exposed all three populations to NaCl in the same environmental conditions, we may not have understood either their genetic predisposition to tolerate NaCl exposure or how they might have responded in their natal lake water environment. Our findings show that gaining a firmer understanding of both the environmental and evolutionary drivers that shape pollution tolerance will improve our ability to reduce pollution impacts and protect ecosystem integrity. Human impacts, including climate change, invasive species, overharvesting, and pollution stress, have been shown to drive greater rates of phenotypic change compared with more “natural” stressors (Hendry et al., 2008). Accounting for adaptation to local environmental conditions when examining the impacts of another stressor may provide greater predictive power in understanding how populations may evolve in response to the selective pressures associated with toxicant exposure and other anthropogenic environmental impacts.

## CONFLICT OF INTEREST STATEMENT

The authors declare no conflicts of interest.

## Supporting information


Appendix S1.

## Data Availability

Reproduction, survival, and *r* data underlying the results presented in Figures 2, 3, 4, 5, 6 are available through the Bowdoin Digital Commons at https://digitalcommons.bowdoin.edu/biology‐faculty‐publications/195/.
