# Supplementary material for: Lake water chemistry and local adaptation shape NaCl toxicity in Daphnia ambigua
Source: Evol Appl. 2024 Mar 22;17(3):e13668. doi: 10.1111/eva.13668 (PMC10960079; doi:10.1111/eva.13668)
Supplement: Supplementary file 1 — Appendix S1. [file EVA-17-e13668-s001.docx]

**List of Supplementary Materials for “**Lake water chemistry and local adaptation shape NaCl toxicity in *Daphnia ambigua”*

1. Supplementary Figure 1. Visualization of the potential influence of lake water environment, population of origin, and NaCl treatment on Daphnia fitness
2. Supplemental text 1. Added calcium mitigates NaCl toxicity in Hall Pond’s low-Ca^2+^ lake water

***
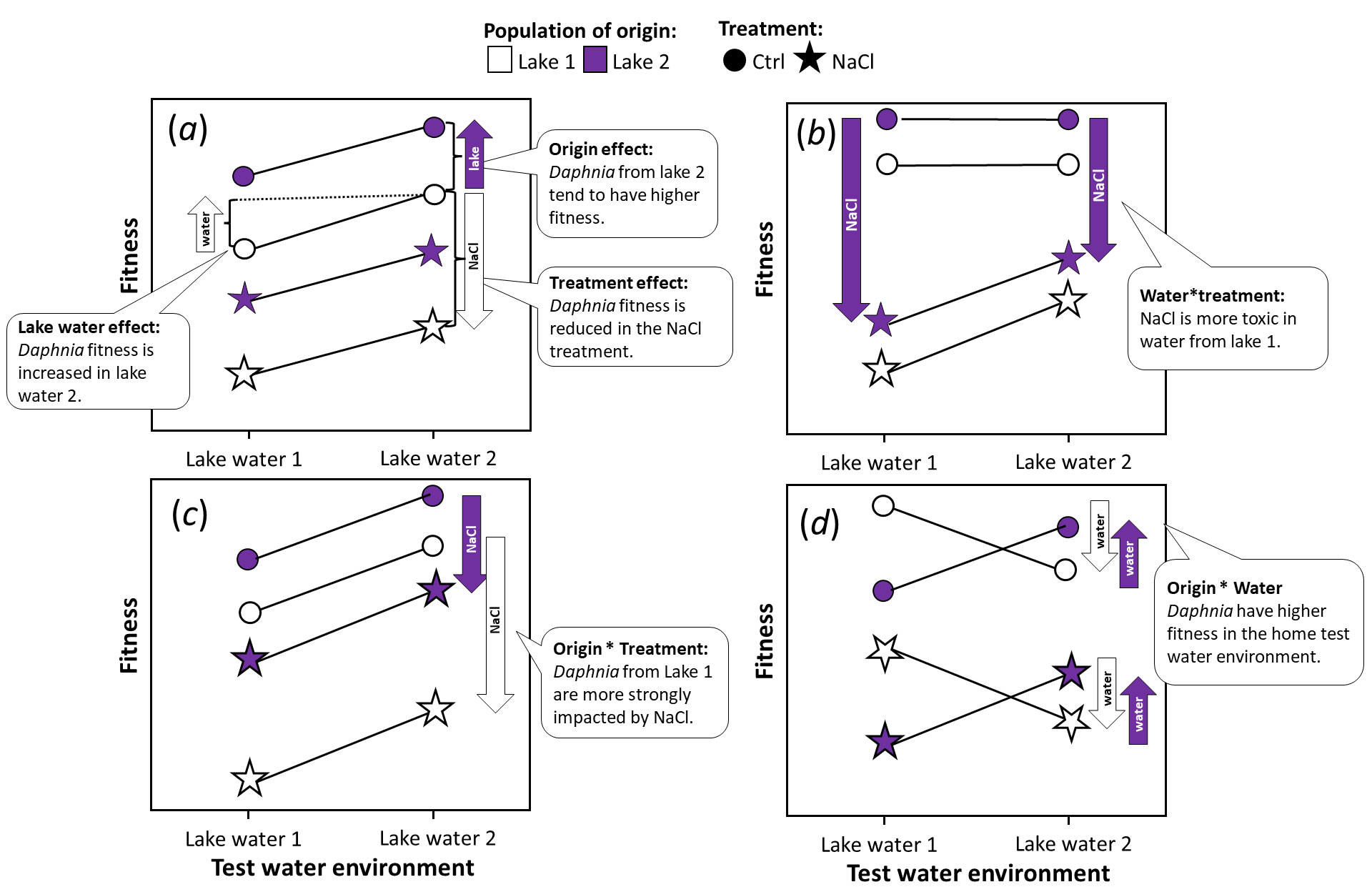
Supplementary Figure 1.*** ***Visualization of the potential influence of lake water environment, population of origin, and NaCl treatment on Daphnia fitness i****n the absence (a) or presence (b,c,d) of interactions among the predictor variables. Colors represent population of origin; shapes represent NaCl treatment (control and + 825 mg/L NaCl); arrows indicate the direction and effect size of predictor variables.*

**Supplemental text 1. Added calcium mitigates NaCl toxicity in Hall Pond’s low-Ca^2+^ lake water**

In a follow up trial conducted in Fall 2021, we examined the influence of added calcium in mitigating NaCl toxicity in Hall Pond’s low-calcium environment. We included six treatments: filtered lake water (1µm glass fiber filters, Pall Corporation) from either Hall Pond or Egypt Pond, filtered Hall Pond water with calcium (in the form of reagent grade CaCl_2_, Fisher Scientific) added to replicate levels present in Egypt Pond (Suppl. Table 2), and a treatment adding 825 mg/L of NaCl (reagent grade, Fisher Scientific) to each of these test media. Measured concentrations of added NaCl were within 4-6.5% of nominal values.

***Supplementary Table 1.*** *Chemical characteristics of control lake water from Hall Pond, Egypt Pond, and Hall Pond with calcium levels adjusted to simulate Egypt Pond water. Mean ion concentrations in mg/L. Major ions were measured with a Dionex Ion Chromatograph ICS-1100 (Thermo Fischer Corporation).*

| **Lake water** | **Na^+^** | **K^+^** | **Mg^2+^** | **Ca^2+^** | **SO_4_^2-^** | **Cl^-^** |
| --- | --- | --- | --- | --- | --- | --- |
| Hall Pond | 2.15 | 0.28 | 0.24 | 1.46 | 0.63 | 3.81 |
| Hall Pond + CaCl_2_ | 2.02 | 0.28 | 0.23 | 6.20 | 0.63 | 11.14 |
| Egypt Pond | 2.75 | 0.47 | 048 | 6.01 | 1.07 | 5.21 |

We exposed 10 replicates of two *Daphnia ambigua* clonal lineages from Hall Pond to each of the six treatments (N=120) for a period of 14 days, using 6-24 hr old neonates to initiate the trial. *Daphnia* lineages were allowed one generation to acclimate to the control test wasters before the trial. We reared *Daphnia* under standard culturing practices described in the main text (individuals reared in 25 mL of filtered lake water, fed 500,000 cells *Ankistrodesmus falcatus* four times weekly, twice weekly water changes, 20˚C 16:8 hr light: dark cycle). We counted and removed offspring and evaluated survival daily.

We evaluated the effect of the lake water environment, NaCl treatment, and their interaction in predicting survival duration, the likelihood of reproduction (binomial response), total offspring produced for those animals that reproduced (zero truncated Poisson distribution), and timing of first reproduction using generalized linear mixed models (GLMMs). Clonal lineage was included as a random intercept. We selected the best fit model for each response variable by comparing the Akaike information criterion (AIC) of the saturated model with nested simpler models containing fewer fixed effects. We used likelihood ratio tests to select which fixed effects to include in the final model, based on Zuur et al. (2009). Analyses were conducted in R (v. 4.2.1) [1]. We used the glmmTMB package [2] to analyze patterns in survival, reproduction timing, and total offspring produced. To examine the likelihood of reproduction, we used the blme package [3] specifying informative priors as recommended by Chung et al. [4] due to the complication of complete separation in the data (100% of individuals reproduced in Egypt Pond water control and NaCl treatments).

We observed an increase in NaCl toxicity in Hall Pond’s low-calcium environment that was very similar to patterns observed in trial 1, reported in the main text. In this follow up trial, the Hall Pond test solution with calcium added to mimic Egypt Pond’s concentrations led to a decrease in NaCl toxicity that was statistically indistinguishable from the toxicity responses observed in Egypt Pond water (Suppl. Fig. 2).


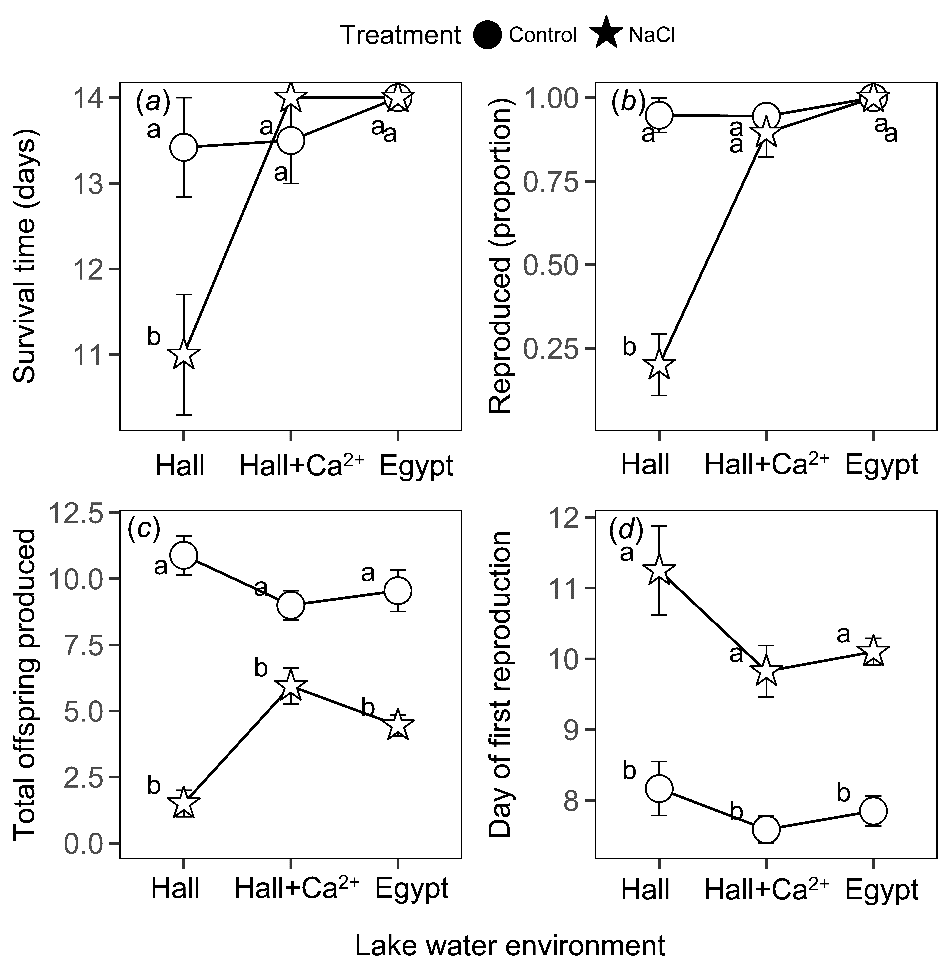
pro

***Supplementary Figure 2.*** *Impacts of added NaCl on survival duration (a), the likelihood of reproduction (b), total offspring produced during the 14-day trial (c), and the timing of first reproduction (d) in three different test media (x-axis): low-calcium Hall Pond water, high-calcium Egypt Pond water, and Hall Pond water with calcium added to equal Egypt Pond concentrations (Supp. Table 1). Circles show control treatments and stars NaCl conditions. a-b designates pairwise differences in post-hoc Tukey tests of GLMM results, where the same letter indicates no significant difference (p>0.05). Note: this figure is also included in the main text as figure 6.*

We observed a significant interaction between the lake water environment and NaCl treatment in predicting survival duration (p=0.001, χ^2^ = 13.55, ΔAIC=-9.56; Suppl. Fig.2*a*), the likelihood of reproduction (p<0.001, χ^2^ = 13.95, ΔAIC=-9.95; Suppl. Fig.2*b*), and total offspring produced (p<0.001, χ^2^ = 20.33, ΔAIC=-16.3; Suppl. Fig.2*c*). NaCl had no effect on survival time or the likelihood of reproduction in both the Egypt Pond and Hall Pond water with added calcium (Control vs. NaCl treatment comparison Tukey HSDs, p>0.961), yet in Hall Pond’s low-calcium water NaCl reduced survival time by 2.4 days (Tukey HSD p=0.001, t ratio=4.07) and led to an 81% decrease in the likelihood of reproduction (Tukey HSD, p=0.001, t-ratio=3.98). For those *Daphnia* that reproduced, NaCl caused an 92% decrease in fecundity in Hall Pond’s lake water but only a 54% decrease in Egypt Pond water and a 35% decrease in Hall Pond water with added calcium (Suppl. Fig.2*c*). Reproduction was delayed in the NaCl treatment (p<0.001, χ^2^ = 59.41, ΔAIC=-57.41), but we observed no effect of the test water (p=0.064, χ^2^ = 5.51, ΔAIC=-1.5) environment or an interaction between test water conditions and NaCl (p=0.4766, χ^2^ = 1.48, ΔAIC=+2.5) in predicting the timing of reproduction (Suppl. Fig.2d).

Together, this suggests that much of the protection against NaCl toxicity in Egypt Pond’s lake water can be explained by elevated calcium levels, a finding well supported by robust laboratory study [5–8].

**References**

1. R Core Team. 2016 *R: A language and environment for statistical computing*. Vienna, Austria: R Foundation for Statistical Computing. See https://www.r-project.org/.

2. Brooks M, Kristensen K, van Benthem K, Magnusson A, Berg C, Nielsen A, Skaug H, Maechler M, Bolker B. 2017 glmmTMB balances speed and flexibility among packages for zero-inflated generalized linear mixed modeling. *R J.* **9**, 378–400.

3. Dorie V, Dorie MV, Maechler M, Bolker B, Walker S. 2021 Package ‘blme’. Bayesian linear mixed model.

4. Chung Y, Rabe-Hesketh S, Dorie V, Gelman A, Liu J. 2013 A nondegenerate penalized likelihood estimator for variance parameters in multilevel models. *Psychometrika* **78**, 685–709.

5. Soucek DJ, Linton TK, Tarr CD, Dickinson A, Wickramanayake N, Delos CG, Cruz LA. 2011 Influence of water hardness and sulfate on the acute toxicity of chloride to sensitive freshwater invertebrates. *Environ. Toxicol. Chem.* **30**, 930–938. (doi:10.1002/etc.454)

6. Mount DR *et al.* 2016 The acute toxicity of major ion salts to *Ceriodaphnia dubia*: I. influence of background water chemistry. *Environ. Toxicol. Chem.* **35**, 3039–3057. (doi:10.1002/etc.3487)

7. Gillis PL. 2011 Assessing the toxicity of sodium chloride to the glochidia of freshwater mussels: Implications for salinization of surface waters. *Environ. Pollut.* **159**, 1702–1708. (doi:10.1016/j.envpol.2011.02.032)

8. Soucek DJ, Mount DR, Dickinson A, Hockett JR. 2018 Influence of dilution water ionic composition on acute major ion toxicity to the mayfly *Neocloeon triangulifer*. *Environ. Toxicol. Chem.* **37**, 1330–1339. (doi:10.1002/etc.4072)
